# Synthetic biology approach for plant protection using dsRNA

**DOI:** 10.1111/pbi.12904

**Published:** 2018-03-25

**Authors:** Annette Niehl, Marjukka Soininen, Minna M. Poranen, Manfred Heinlein

**Affiliations:** ^1^ Université de Strasbourg CNRS IBMP UPR 2357 Strasbourg France; ^2^ Molecular and Integrative Biosciences Research Programme Faculty of Biological and Environmental Sciences University of Helsinki Helsinki Finland; ^3^ Present address: Julius Kühn‐Institute (JKI) Braunschweig Germany

**Keywords:** RNAi, dsRNA production technology, sustainable crop protection

## Abstract

Pathogens induce severe damages on cultivated plants and represent a serious threat to global food security. Emerging strategies for crop protection involve the external treatment of plants with double‐stranded (ds)RNA to trigger RNA interference. However, applying this technology in greenhouses and fields depends on dsRNA quality, stability and efficient large‐scale production. Using components of the bacteriophage phi6, we engineered a stable and accurate *in vivo* dsRNA production system in *Pseudomonas syringae* bacteria. Unlike other *in vitro* or *in vivo* dsRNA production systems that rely on DNA transcription and postsynthetic alignment of single‐stranded RNA molecules, the phi6 system is based on the replication of dsRNA by an RNA‐dependent RNA polymerase, thus allowing production of high‐quality, long dsRNA molecules. The phi6 replication complex was reprogrammed to multiply dsRNA sequences homologous to tobacco mosaic virus (TMV) by replacing the coding regions within two of the three phi6 genome segments with TMV sequences and introduction of these constructs into *P. syringae* together with the third phi6 segment, which encodes the components of the phi6 replication complex. The stable production of TMV dsRNA was achieved by combining all the three phi6 genome segments and by maintaining the natural dsRNA sizes and sequence elements required for efficient replication and packaging of the segments. The produced TMV‐derived dsRNAs inhibited TMV propagation when applied to infected *Nicotiana benthamiana* plants. The established dsRNA production system enables the broad application of dsRNA molecules as an efficient, highly flexible, nontransgenic and environmentally friendly approach for protecting crops against viruses and other pathogens.

## Introduction

Agricultural yields are greatly affected by various pathogens and pests which decrease production of crops worldwide (Oerke, 2005) with losses adding up to around $US100 billion per year (Fletcher *et al*., 2006) and potentially reaching U$540 billion per year if the spreading of invasive pests and pathogens is not controlled (Kew Royal Botanical Gardens, 2017; Paini *et al*., 2016). To protect plants against pathogens and pests, agriculture relies on the widespread use of chemical pesticides that are applied to the environment in large amounts. These intense applications of chemical pesticides pose potential risks of human health, beneficial organisms and the environment (Nicolopoulou‐Stamati *et al*., 2016). Thus, there is a need for novel tools and alternative methods to control disease epidemics. Indeed, legislation in Europe is aiming at reducing the use of chemical plant protection products and calling for an in‐depth reconsideration of crop protection solutions (Union, 2009). A promising new approach with strong potential for protecting plants against viruses and other pathogens involves the application of double‐stranded (ds)RNA.

dsRNA is a natural product that occurs in most, if not all, organisms in nature. It triggers RNA silencing, also known as RNA interference (RNAi), which is a conserved eukaryotic mechanism playing a crucial role in growth, development, host defence against viruses and transposon inactivation, across plant, fungi and animal kingdoms. RNAi plays a natural role in antiviral defence by targeting the invading viral RNA genomes and viral transcripts for cleavage and translational repression in a sequence‐specific manner (Ding, 2010; Pumplin and Voinnet, 2013). Consistent with the central role of dsRNA in triggering RNAi, numerous studies have shown that plants treated with virus‐specific dsRNA or single‐stranded RNA (ssRNA) molecules that fold into hairpin conformation showed antiviral resistance (Carbonell *et al*., 2008; Gan *et al*., 2010; Konakalla *et al*., 2016; Tenllado and Diaz‐Ruiz, 2001; Tenllado *et al*., 2003a,b; Yin *et al*., 2009). dsRNAs were also shown to be effective against fungi (Koch *et al*., 2016; Wang *et al*., 2016, 2017) and insects (Baum *et al*., 2007; Ghosh *et al*., 2017; Li *et al*., 2013; Luo *et al*., 2017), thus providing important implications for dsRNA‐triggered RNAi as an emerging novel approach for crop protection. RNAi has been deployed for plant protection since it was discovered. A plethora of studies has demonstrated the feasibility of RNAi‐based approaches, and numerous virus‐resistant crops have been approved for commercial release (Khalid *et al*., 2017). However, unlike these previous approaches, which depend on transgenes, the external application of dsRNA will realize crop protection without any modification of the plant genome and, therefore, may be regulated differently compared to crop protection involving genetically modified organisms. Moreover, in being independent of any time‐consuming genetic modification, the application of dsRNA is much more flexible and allows faster actions against new emerging diseases.

Applying dsRNA may have numerous advantages over the use of chemical compounds. Unlike chemical compounds, which act via a structure‐dependent mechanism, dsRNAs act by means of their specific nucleotide sequence. Thus, in being designed to act against a specific pathogen target with homologous sequence, the dsRNA agent and derived small interfering (si)RNAs are expected to act only against the anticipated pathogen. As dsRNA activity depends on plant RNAi pathways that are essential for plant development, and given that dsRNA molecules are designed to target the pathogen and not the plant, the occurrence of plant mutations leading to resistance against dsRNA‐triggered RNAi is unlikely. Moreover, as dsRNAs exert their inhibitory mode of action throughout their entire sequence length, the evolution of pathogen resistance by selection of dsRNA target sequence mutations is fairly unlikely as well. Importantly, in contrast to chemical pesticides, dsRNA agents are biocompatible and biodegradable compounds that are part of nature and occur ubiquitously inside and outside organisms as well as in food. Like any RNA molecule, also dsRNA shows low stability in water or soil (Dubelman *et al*., 2014). Within plants and other organisms, dsRNAs enter natural RNA silencing pathways and are degraded to small RNAs, which themselves are subject to turnover through natural degradation mechanisms (Cerutti and Ibrahim, 2010). Thus, RNA sprays will not produce any substantially novel residues in food products.

However, the broad application of dsRNA treatments in greenhouses and fields is hampered by the lack of efficient and economical methods for dsRNA design, large‐scale production and purification. The main approach for producing dsRNAs has so far been physical annealing of two enzymatically synthesized ssRNA strands. Annealing is performed either *in vitro* (Carbonell *et al*., 2008; Koch *et al*., 2016; Konakalla *et al*., 2016; Tenllado and Diaz‐Ruiz, 2001; Wang *et al*., 2016) or *in vivo* following ssRNA synthesis in RNase III‐deficient bacterial cells (Gan *et al*., 2010; Tenllado *et al*., 2003a; Yin *et al*., 2009). Even though dsRNA can be produced by these methods, the physical hybridization of two complementary ssRNA molecules *in vitro*, and especially *in vivo*, often results in relatively low yields of correctly and fully duplexed dsRNA. Moreover, the bacterial production systems contain homologous DNA molecules, which affect the quality of the RNA preparation and its applicability. A more biological and accurate method to produce dsRNA is to utilize enzymes encoded by dsRNA viruses that are specialized in the synthesis of dsRNA. The RNA‐dependent RNA polymerase of the dsRNA phage phi6 (Makeyev and Bamford, 2000) converts ssRNA templates into dsRNA with high processivity using a *de novo*, primer‐independent initiation mechanism (Laurila *et al*., 2002). In pioneering work, we previously explored the possibility to use components of phi6 for the *in vivo* synthesis of dsRNA aimed for subsequent Dicer digestion and use in animal cell cultures (Aalto *et al*., 2007). However, the system proved to be unstable for the efficient production of most dsRNA molecules. Here, we report the engineering of a stable and accurate *in vivo* dsRNA replication system in bacteria that allows the large‐scale production of long dsRNA molecules targeting pathogen genes or genomes and suitable for application in crop protection.

## Results

**Figure 1:**
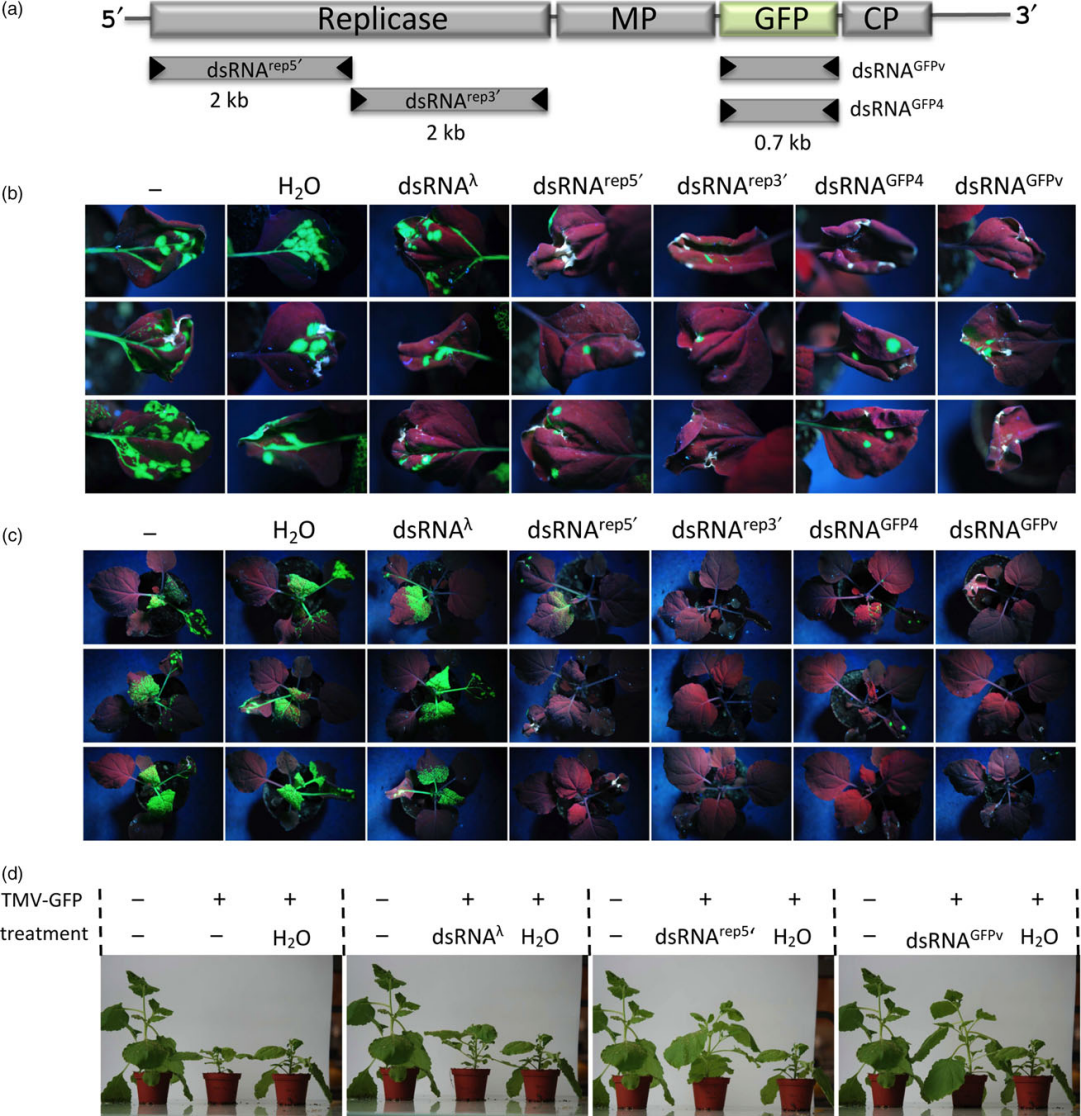
Effect of in vitro‐produced dsRNAs on the propagation of TMV‐GFP and associated disease symptoms in *N. benthamiana* plants. (a) RNA genome of TMV‐GFP with highlighted protein‐coding genes and derived dsRNAs produced *in vitro* (MP, movement protein; CP, capsid protein). The sequence of dsRNAGFP4 corresponds to a specific GFP variant, GFP4 (Haseloff *et al*., 1997) that differs from the virus‐encoded GFP (GFPv) by 9% at the nucleic acid level. (b and c) State of infection at 7 dpi. (b) Inoculated leaves. (c) Whole plants. Single leaves of plants were inoculated with TMV‐GFP either alone, or together with H_2_O or *in vitro*‐produced nonspecific control dsRNA (dsRNA ^λ^), or dsRNA ^rep5′^, dsRNA ^rep3′^, dsRNA ^GFP4^ or dsRNA ^GFPv^. Three plants per treatment are shown. Photographs were taken under ultraviolet (UV) light. Infected leaf areas show green fluorescence and noninfected leaf areas are red fluorescent (chlorophyll autofluorescence). (d) Symptom phenotypes of treated plants at 14 dpi. Representative images are shown. The individual image panels show the same healthy noninoculated control plant on the left and the same diseased TMV‐GFP infected, H_2_O‐treated control plant on the right. A TMV‐GFP infected and untreated or dsRNA‐treated plant is in the middle of each panel as indicated. The experiment was performed two times with three to four plants per condition.

For the initial assessment of the effects of dsRNA vaccination on virus infection, we used target‐specific dsRNA molecules that were produced *in vitro* using DNA‐dependent T7 RNA polymerase for DNA transcription and the RNA‐dependent RNA polymerase of phi6 for subsequent RNA replication (Figure S1). The dsRNAs were applied to *Nicotiana benthamiana* plants infected with a GFP‐tagged tobacco mosaic virus (TMV‐GFP) which allows the *in vivo* monitoring of infection by the analysis of GFP fluorescence. Treating the plants with different *in vitro*‐produced dsRNA molecules targeting different parts of the viral replicase gene or the GFP gene inserted in the genome of the TMV‐GFP virus led to efficient inhibition of viral propagation in the treated plants (Figure 1, Tables S1, and S2). However, producing such efficient dsRNAs with purified enzymes will not be feasible for large‐scale applications. Thus, to develop this approach further and to particularly prepare this technology for large‐scale applications, we engineered a phi6‐based dsRNA‐synthesizing machine within bacteria.

**Figure 2:**
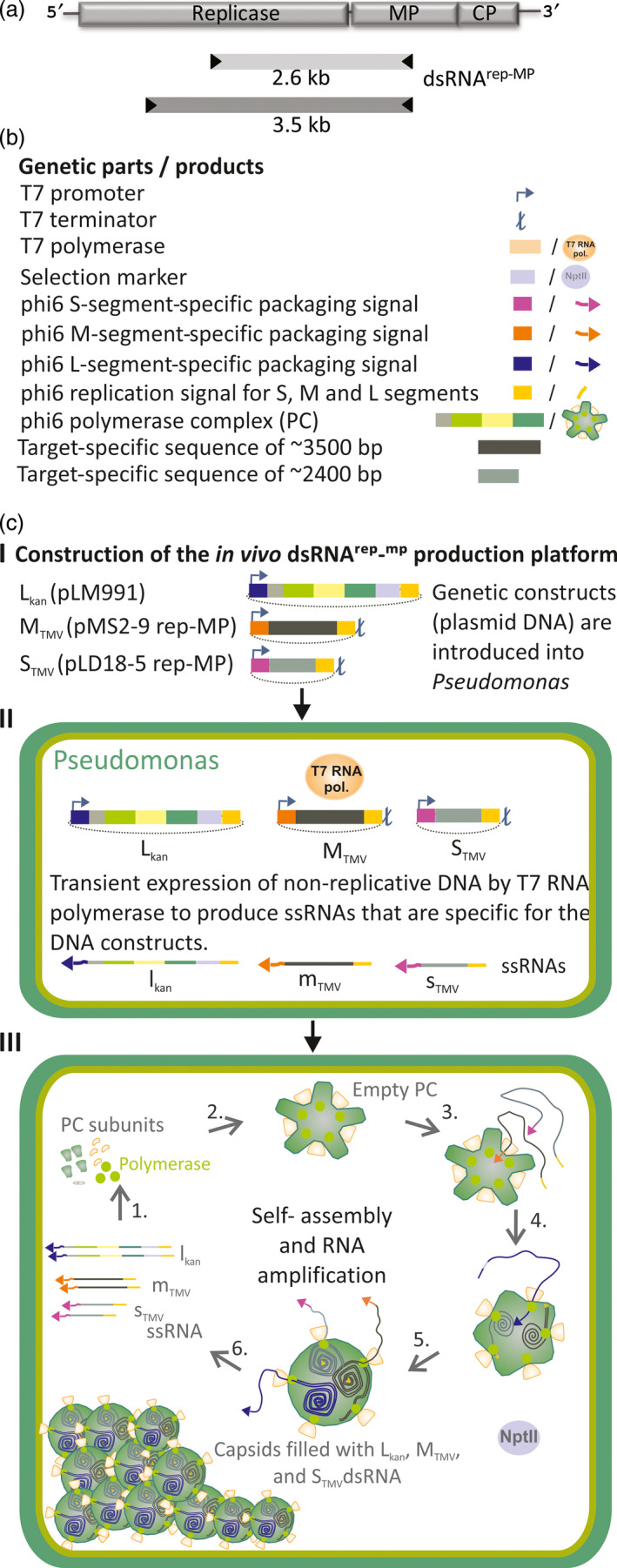
Design and construction of the phi6 *in vivo* dsRNA production platform. (a) Schematic illustration of the RNA genome of TMV and the areas selected for *in vivo* dsRNA production. (b) General genetic components for the construction of a stable *in vivo* dsRNA production platform. These originate from (i) the phi6 S, M and L genomic segments, (ii) T7 bacteriophage, and (iii) target (here: TMV). Optimal sizes of target‐specific sequences, which should be used to produce dsRNA molecules corresponding to the natural sizes of the S and M genome segments, are indicated. (c) Construction of the *in vivo* dsRNA ^rep‐^ ^MP^ production platform. (c, I) Constructs for *P. syringae* transformation. L_kan_ encodes the components of the phi6 polymerase complex (PC), L‐segment‐specific RNA replication and packaging signals and the kanamycin resistance gene *nptI*. S_TMV_ and M_TMV_ contain TMV‐specific sequences (without open reading frames) flanked by phi6 S‐ or M‐segment‐specific signal sequences, respectively. Each construct carries a T7 RNA polymerase promoter upstream of the segment‐specific phi6 packaging signal. (c, II) Transformation of the plasmids into *P. syringae* cells, which express T7 RNA polymerase, leads to the transcription of L_kan_‐, M_TMV_ ‐ and S_TMV_ ‐specific ssRNA molecules (l_kan_, m_TMV_ and s_TMV_ ). T7 terminators ensure correct length of the M_TMV_ ‐, and S_TMV_ ‐specific ssRNA transcripts. (c, III) Translation of l_kan_ ssRNA (1.) leads to production of empty PCs (2.), which are sequentially packaged with s_TMV_ , m_TMV_ and l_kan_ ssRNAs (3.). During packaging, the PC synthesizes the complementary strand for each packaged ssRNA carrying the phi6‐specific RNA replication signal (4.). Following the first round of dsRNA synthesis, the PCs continue to synthetize more s_TMV_ , m_TMV_ and l_kan_ ssRNAs (6.), which lead to the amplification of PCs containing L_kan_, M_TMV_ and S_TMV_ dsRNAs (dsRNA ^rep‐^ ^MP^ ) until the host cell is filled with such particles. The plasmids used for the initial transformation (c, I−II) do not replicate in *P. syringae* and are not present in the cell lines in which dsRNA production is established (III).

**Figure 3:**
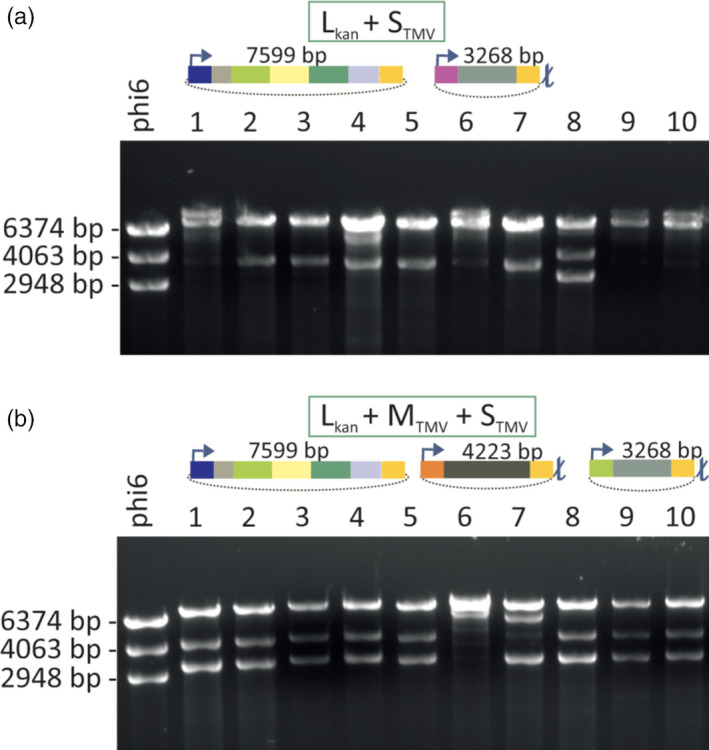
Genetic stability of the dsRNA production cell lines. *P. syringae* cells were transformed with L_kan_ construct together with (a) S_TMV_ construct, or (b) S_TMV_ and M_TMV_ constructs. Kanamycin‐resistant cell lines were selected from each transformation and further cultivated. dsRNA was isolated from the different bacterial cell cultures and analysed by agarose gel electrophoresis (dsRNAs produced in 10 cell lines are shown). Phi6 genomic dsRNA composed of the L (6374 bp), M (4063 bp) and S (2948 bp) dsRNA molecules was used as a size marker (left lane). The DNA constructs used for the transformations are schematically depicted above the gel images. A dsRNA molecule corresponding to the size of the L_kan_ dsRNA (7599 bp) is present in all transformants (a and b). However, only few of the cell lines in (a) replicate dsRNA that would correspond the size of S_TMV_ (3268 bp). In contrast, 80% of the cell lines originating from the transformation with L_kan_, S_TMV_ and M_TMV_ constructs (b) produced expected dsRNA molecules (7599, 4223 and 3268 bp, respectively).

The genome of phi6 is composed of three dsRNA segments termed ‘S’ (2948 bp), ‘M’ (4063 bp) and ‘L’ (6374 bp). Upon infection, the polymerase complex (PC) that forms the internal layer of the phi6 phage particle is delivered to the cytoplasm of the host bacterium. The polymerase subunits residing inside the PC then starts the synthesis of phage ssRNAs using the encapsidated genomic dsRNA molecules as templates. These ssRNAs (s, m and l) are delivered into the cytoplasm whereby the L‐segment‐specific ssRNAs direct the synthesis of proteins that self‐assemble into new PCs that are subsequently filled with the ssRNAs. After completion of the ssRNA packaging, the polymerase subunits within the PC are activated and synthetize the complementary strands for each encapsidated ssRNA segment (Figure S2). To generate specific dsRNA‐producing bacteria, we first assembled an S‐segment‐specific construct, S_TMV_ (Figure 2, Table S3), in which we incorporated sequences derived from TMV. Assuming that the size of the phi6 segments may be critical for dsRNA production, the length of the S_TMV_ dsRNA was designed to match the size of the natural phi6 S‐segment. Thus, the TMV‐derived sequence that we inserted between the S‐segment‐specific replication and encapsidation signals was 2611 nts in length and covered parts of the TMV replicase and movement protein (MP) genes (Figure 2a). To ensure that the ssRNAs initially transcribed from the plasmid is terminated at the correct length, a T7 terminator was inserted downstream the S_TMV_ construct in the corresponding plasmid vector (pLD18‐5 rep‐MP; Figure 2b,c). However, *P. syringae* cells transformed with this vector together with a vector encoding a modified phi6 L‐segment carrying a selectable marker gene (L_kan_) failed to stably produce S_TMV_ dsRNA of expected size (3268 bp) (Figure 3a). This failure to produce the expected dsRNA molecules likely relates to the lack of M‐segment‐specific signal sequences that are essential for the normal regulation of genome packaging and replication by the phi6 replication complex (Figure S2). Indeed, genetic stability was achieved when an M‐segment‐specific construct, M_TMV_, was included in the system. Our construct contained a 3540 nts long insert from the TMV genome (Figure 2a) cloned between the phi6 M‐segment‐specific replication and encapsidation signals (Figure 2b,c). Moreover, similar as for the S_TMV_ construct, a T7 terminator sequence was cloned downstream the M_TMV_ construct (4223 bp) in the corresponding plasmid vector (pMS2‐9 rep‐MP) to facilitate the production of correct‐length RNA.

Transformation of *P. syringae* cells with vectors containing the L_kan_, M_TMV_ and S_TMV_ constructs led to efficient recovery of transformed cell lines expressing the expected dsRNA pattern (7599, 3268 and 4223 bp, respectively; Figure 3b). Moreover, in addition to the increased stability, the inclusion of the M‐segment‐specific construct substantially increased the amount of target‐specific dsRNA that could be replicated within the phi6 PCs. Our dsRNA preparation, dsRNA^rep‐MP^, contained L_kan_, S_TMV_ and M_TMV_ dsRNAs in equal ratios and the system produced about 700 μg dsRNA^rep‐MP^ per 100 ml culture (4 × 10^9^ cells/ml). In addition to the TMV‐specific dsRNA‐producing cell lines, we also generated a control cell line (transformed with plasmids pLM991, pLM656 and pMH4) that replicates phi6‐specific L_kan_, M and S_lys_ dsRNAs (dsRNA^phi6^) without any foreign inserts except the selection marker gene (Table S3). S_lys_ is a S‐segment that is wild type except for a mutation that inactivates *gene 5* and thereby prevents the expression of the phi6 lytic functions (Sarin *et al*., 2012).

**Figure 4:**
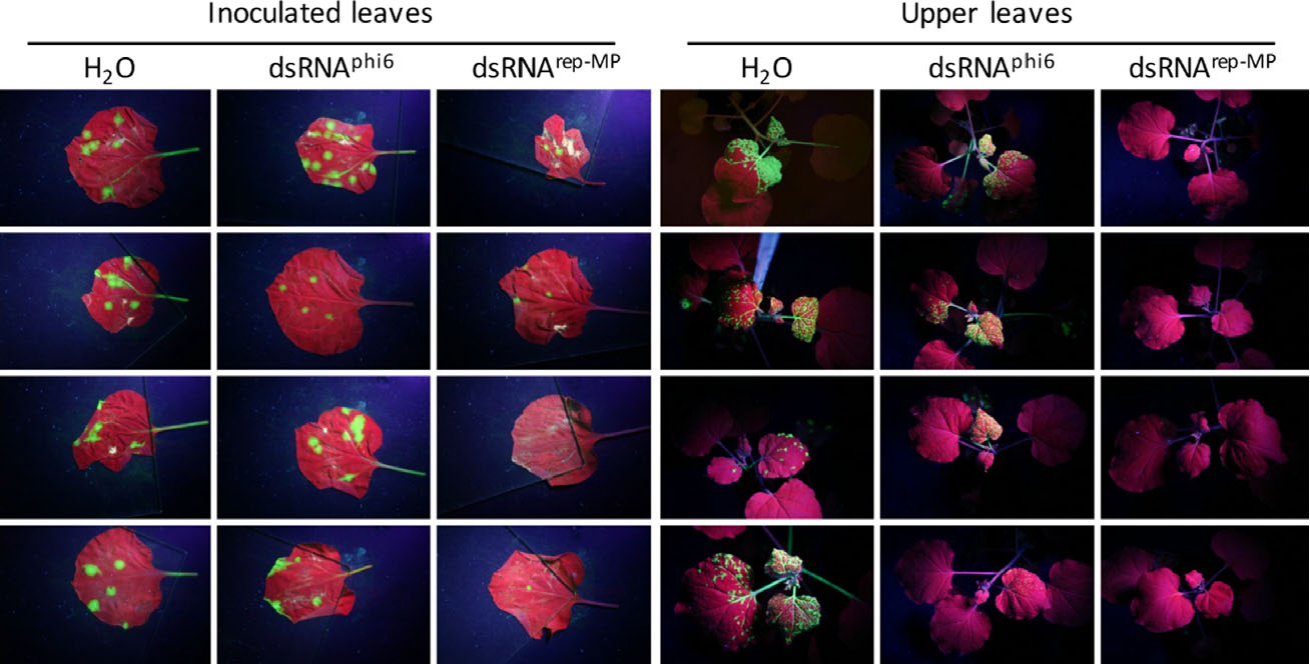
Effect of *in vivo*‐produced dsRNAs on the local and systemic spread of TMV‐GFP in infected *N. benthamiana* plants. Single leaves of plants were mechanically inoculated with TMV‐GFP together with either H_2_O, purified dsRNA ^phi6^ or purified dsRNA ^rep‐^ ^MP^ . Four plants per treatment are shown. Unlike treatment with water or dsRNA ^phi6^, the treatment with dsRNA ^rep‐^ ^MP^ inhibits the local and systemic spread of the virus. Photographs were taken at 14 dpi under UV light. Infected leaf areas show green fluorescence, and noninfected leaf areas are red fluorescent (chlorophyll autofluorescence).

**Figure 5:**
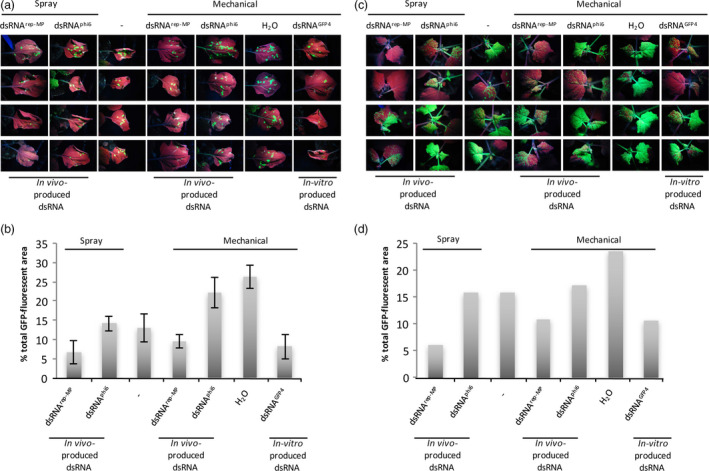
Effect of local dsRNA treatment by spraying or mechanical inoculation on the local and systemic spread of TMV‐GFP in infected *N. benthamiana* plants. The virus‐inoculated leaves were cotreated with *in vivo*‐produced and purified dsRNA ^rep‐^ ^MP^ or dsRNA ^phi6^ by either spraying or mechanical inoculation, as indicated. The virus‐inoculated leaves of control plants were either not treated (spraying control indicated with dash) or mechanically inoculated with either H_2_O or *in vitro*‐produced dsRNA^GFP^ ^4^ (control for mechanical treatment). (a) Virus‐inoculated leaves at 7 dpi. Four plants per treatment are shown. Photographs were taken under UV light. Some plants were also part of the time‐course experiment shown in Figure S3 (0‐day time point). (b) Quantitative analysis of the relative sizes of the infected leaf areas per condition. The total GFP fluorescent area measured for all the leaves was set to 100%, and the distribution of the fluorescence between the conditions was quantified from the image shown in (a). Similar quantitative analysis was also performed at 9 dpi and 14 dpi. The columns show the mean value and standard deviation for these three individual measurements. (c) Infection in the systemic, nontreated leaves of same plants as in (a) at 9 dpi. (d) Quantitative analysis of the relative sizes of the infected GFP fluorescent leaf areas per condition seen in (c).

Applying purified *in vivo*‐produced dsRNA^rep‐MP^ (L_kan_, M_TMV_ and S_TMV_ dsRNAs) either mechanically or by spraying to the virus‐inoculated leaves provided efficient protection against local spread of TMV (Figures 4 and 5). Consistent with the inhibition of virus spread at the local level, the dsRNA treatment also reduced the systemic spread of the virus, although to variable extent (Figure 4). The protective effect of our dsRNA remained stable for at least 7 days when sprayed onto the leaves (Figure S3).

## Discussion

The *in vivo* dsRNA production machine we have engineered and tested here is easily adaptable to different target sequences and, therefore, has great potential to permit therapeutic dsRNAs to be designed and produced for large‐scale crop protection against different viral and fungal pathogens, and insect pests. The very long dsRNA molecules (>2600 base pairs) produced by this system give rise to a large pool of target‐specific siRNAs. The presence of such a large pool of siRNAs for a single target (i.e. a viral genome) increases target specificity and, unlike strategies involving single or few target‐specific siRNAs, avoids the evolution of mutations in the target by which the targeted organism may become resistant to the treatment. The ability to produce long dsRNAs also offers the possibility of pyramidic plant protection approaches in which dsRNAs with sequence homology to multiple and diverse targets are fused to protect plants against several pathogens simultaneously.

The major technical obstacle for the utilization of the phi6‐based *in vivo* dsRNA production system for heterologous dsRNA production (Aalto *et al*., 2007) has been the compromised genetic stability of the cell lines produced. The lack of the M‐segment in the previous design has led to unpredictable recombination events, instability and repeated failure to produce the expected dsRNA, similar to that observed when we attempted to produce cell lines replicating only L_kan_ and S_TMV_ dsRNAs (Figure 3A). Based on the current knowledge about the regulation of phi6 dsRNA replication (Figure S2), this challenge was overcome here (Figure 3B) by including the M‐segment and other essential regulatory elements from the phi6 genome (Figure 2). Using the M‐segment in addition to the S‐segment not only increased stability but also substantially increased the amount of target‐specific dsRNAs produced by the phi6 polymerase complex. The incorporation of the M‐segment into the dsRNA production system also may facilitate the development of pyramidic plant protection approaches by producing M‐ and S‐segments that generate different dsRNAs against different pathogen targets.

*P. syringae* cell lines harbouring the engineered dsRNA production of interest can be grown in fermenters, which opens the way for large‐scale vaccination applications to protect plant crops against a variety of important diseases. Important complementary new technology has been conceived to enhance the stability of dsRNA upon spraying and prolonging the time frame for plant protection to 20 days or more (Mitter *et al*., 2017). Therefore, the topical application of dsRNAs has great potential to become a novel and widespread environmentally friendly disease management tool that circumvents technical limitations of plant transformation and the public concerns about genetically modified organisms.

## Experimental procedures

### Bacterial strains and viruses

*P. syringae* strain LM2691 (Sun *et al*., 2004) was used as a host for phi6‐based dsRNA production. This strain harbours plasmid pLM1086 (Sun *et al*., 2004) that constitutively expresses T7 RNA polymerase. *Escherichia coli* strain DH5α was used as host for the production of recombinant plasmids. Virion particles of green fluorescent protein‐tagged TMV (TMV‐GFP) (Lindbo, 2007) were produced by infection of *N. benthamiana* plants and by applying a TMV purification protocol described previously (Niehl *et al*., 2015).

### Oligonucleotides and plasmids

All oligonucleotide primers are summarized in Table S4. An agroinfection‐compatible infectious TMV‐GFP clone [(pJL24 (Lindbo, 2007)] was used as a template for the *in vitro* production of TMV‐ and GFP‐specific dsRNAs, as donor for a viral genome fragment (rep‐MP) described below, as well as for initial infection of *N. benthamiana* plants to produce virion particles for subsequent infection experiments. Plasmid pYY00 (Haseloff *et al*., 1997) was used as a template for *in vitro* production of GFP4‐specific dsRNAs. The phi6 *in vivo* dsRNA production system is based on plasmids pMH4, pLM656 and pLM991, which contain complementary DNA (cDNA) copies of the phi6 genome segments S, M and L, respectively, inserted into plasmid pT7T319U under the T7 RNA polymerase promoter (Olkkonen *et al*., 1990; Sarin *et al*., 2012; Sun *et al*., 2004). The *gene 5* of the S‐segment in the S_lys_ construct encoded by pMH4 contains three successive stop codons preventing the expression of the phi6 lysis functions (Sarin *et al*., 2012). pMH4 also harbours a T7 transcription termination signal downstream the S_lys_‐encoding sequence. The L‐segment encoded by plasmid pLM991 (L_kan_) contains a kanamycin resistance gene (*nptII*) (Sun *et al*., 2004) for antibiotic selection.

The S‐segment‐specific dsRNA production vector pLD18 is a derivative of plasmid pMH4 and was generated by replacing the *Sal*I–*Ksp*I fragment of the S‐segment‐specific cDNA with a 441‐nt long *Sal*I–*Ksp*I fragment containing a multiple cloning site (MCS) from plasmid pPS9(+). pPS9(+) is a derivative of pPS9 (Aalto *et al*., 2007), in which the *egfp* gene‐containing *Eag*I‐*Pst*I fragment was replaced with a 98‐nt long MCS sequence constructed by annealing two complementary DNA oligonucleotides and digested with *Eag*I and *Pst*I. The M‐segment‐specific vector pMS1 was obtained from pLM656 by replacing the *Afl*II–*Pst*I fragment of the M‐segment in pLM656 with a 98‐nt long MCS with complementary cohesive ends. The MCS sequences were constructed by annealing two complementary DNA oligonucleotides. The dsRNA production vector pMS2 was obtained from pMS1 by introducing the termination signal sequence for T7 polymerase [TATCTGTTGTTTGTCG (Sohn and Kang, 2005)] downstream of the RNA replication signal of the remaining M‐segment cDNA using PCR. Fragments of the TMV‐GFP genome [nucleotides 2850–5460 (2611 nts) and 1921–5460 (3540 nts)] encompassing parts of the replicase and movement protein (MP) sequences (rep‐MP) were cleaved using *Nde*I and *Apa*I, and *Nhe*I and *Apa*I restriction sites, respectively, and were inserted into the *Nde*I–*Apa*I and *Nhe*I–*Apa*I sites within the MCS of pLD18 and pMS2 to produce plasmids pLD18‐5 rep‐MP and pMS2‐9 rep‐MP (Table S3), respectively.

### *In vitro* dsRNA production

dsRNA^rep5′^, dsRNA^rep3′^, dsRNA^GFPv^, dsRNA^GFP4^ and dsRNA^λ^ were produced in *in vitro* using the Replikator RNAi Kit (Finnzymes, Vantaa, Finland; Figure S1) according to the manufacturer's instructions. The DNA templates for dsRNA synthesis were produced by PCR using gene‐specific primers fused to T7 and phi6 promoter sequences (Table S4) and purified using Nucleospin PCR Gel and PCR cleanup columns (Macherey‐Nagel) before use. Following synthesis at 35 °C, the dsRNA preparations were stored at −20 °C until use.

### *In vivo* dsRNA production

For *in vivo* dsRNA production, electroporation‐competent cells of *P. syringae* LM2691 were produced as previously described (Sun *et al*., 2004) and transformed with plasmid pLM991 together with the S‐, or S‐ and M‐segment‐specific constructs (Table S3). After electroporation, the cells were recovered by incubation in SOC solution (2% Bacto tryptone, 0.5% Bacto yeast extract, 10 mm NaCl, 2.5 mm KCl, 10 mm MgCl_2_, 10 mm MgSO_4,_ 20 mm glucose) at +28 °C for 2 h, and the transformed cells were selected by plating them on Luria plates containing kanamycin. The plates were incubated at +28 °C for 2 days.

dsRNA was extracted from concentrated suspensions of overnight grown liquid cultures using TRIzol‐chloroform extraction, stepwise LiCl precipitation, followed by ammonium acetate precipitation. The dsRNA pellet was solved in milliQ‐purified water and stored at ‐20 °C prior application to plant leaves. The concentration of the purified *in vivo‐*produced dsRNA was measured using a NanoDrop 2000 UV‐Vis spectrophotometer.

### Inoculation of plants with virus and dsRNA

*N. benthamiana* plants were grown from seeds on soil in growth chambers with 16‐h/8‐h light/dark periods at +22 °C/+18 °C. Four‐ to seven‐week‐old *N. benthamiana* plants were treated with dsRNA by dusting the leaves with celite and gently rubbing the dusted leaves with approximately 5 μg dsRNA. The same amount of dsRNA was applied for inoculation by spraying using a clean spray bottle (perfume dispenser). Wetting the foliage during watering was avoided during the duration of the experiment. Viruses (20 ng of purified TMV‐GFP virions) were applied by rubbing the dsRNA‐treated leaves in the presence of celite either immediately or at later times after application of dsRNAs, as indicated in the figure legends. Each dsRNA treatment experiment was performed with three to four plants (replicates).

All inoculation experiments involved the use of the same virion preparation to preserve stable inoculation conditions and the same inoculum quality throughout experiments. Moreover, using virions rather than *in vitro*‐transcribed infectious TMV‐GFP RNA as inoculum, we aimed to mimic the conditions of natural virus transmission. Indeed, TMV produces very stable virions that do not depend on insect vectors for interplant transmission and are transmitted mechanically. Although the efficiency of our mechanical inoculation procedure is not known, the applied virion dosage per leaf [ca. 3 × 10^8^ particles, according to a molecular weight of TMV (40 MDa, Santos and Castanho, 1996)] was considerably higher than the number of virions that are transferred to the new host plant by natural contact transmission. Usually, the latter is in the range of single virions (between 1 and 4) despite that the transmitting leaves may contain virus titers as high as 10^7^–10^9^ (Sacristan *et al*., 2011).

### Imaging analysis of plants

*Nicotiana benthamiana* plants were photographed with a Nikon D80 camera and 60‐mm lens between 7 and 14 days postinoculation (dpi), as indicated in the text. For image acquisition, the camera was fixed on a stand in a dark room, and the plants were illuminated using a hand‐held UVP Blak‐Ray B‐100 ultraviolet (UV) lamp. Images were acquired in automatic mode. For imaging of inoculated leaves, the leaves were detached from the plant and flattened (if necessary) by covering them with a glass plate before photographs were taken.

### Quantification of TMV spread and dsRNA vaccination effects

TMV spread within inoculated plants (data in Tables S1 and S2) was quantified by analysis of the GFP‐tagged areas of infection using ImageJ image analysis software (https://imagej.nih.gov/ij/). Using the threshold function of this software, the total area of the green fluorescence in leaves or whole plants was measured and the area in pixels calculated. Mean values and standard deviations (SD) were calculated for each condition and each experiment. Unpaired t‐tests were performed where applicable to test the significance of observations between different samples.

For the analysis of TMV spread in time‐course experiments after mechanical and spray inoculation of *in vivo‐* or *in vitro‐*produced dsRNA (Figures 5 and S3), photographs of all individual plants belonging to an experiment were assembled so that all plant replicates were aligned vertically and all conditions aligned horizontally. The assembled image showing all these plant samples together was thresholded for GFP fluorescence. The green pixel intensity values measured in the vertically aligned replicates for each condition were plotted along the horizontal axis and resulted in green pixel intensity curves, each representing the specific GFP fluorescence values across the four biological replicates for each condition. The area below the curve for each specific condition was integrated and taken as quantitative value for the relative (%) total GFP fluorescence in the replicate plant samples of the specific treatment (total GFP fluorescence in the whole image = 100%).

## Supporting information


**Figure S1** *In vitro* production of dsRNA.
**Figure S2** The phi6 lifecycle.
**Figure S3** Duration of the vaccination effect after spraying or mechanical inoculation of the dsRNAs.
**Table S1** Effect of different dsRNAs compared to water on TMV‐GFP infection in infected leaves.
**Table S2** Effect of different dsRNAs compared to water on the systemic spread of TMV‐GFP.
**Table S3** Plasmids used for the transformation of *P. syringae*.
**Table S4** PCR‐primers and other oligonucleotides.
