# Supplementary material for: Synthetic biology approach for plant protection using dsRNA
Source: Plant Biotechnol J. 2018 Mar 25;16(9):1679–87. doi: 10.1111/pbi.12904 (PMC6097125; doi:10.1111/pbi.12904)
Supplement: Supplementary file 1 — Figure S1 In vitro production of dsRNA. Figure S2 The phi6 lifecycle. Figure S3 Duration of the vaccination effect after spraying or mechanical inoculation of the dsRNAs. Table S1 Effect of different dsRNAs compared to water on TMV‐GFP infection in infected leaves. Table S2 Effect of different dsRNAs compared to water on the systemic spread of TMV‐GFP. Table S3 Plasmids used for the transformation of P. syringae. Table S4 PCR‐primers and other oligonucleotides. [file PBI-16-1679-s001.docx]

**Synthetic biology approach for plant protection using dsRNA**

Annette Niehl, Marjukka Soininen, Minna M. Poranen, Manfred Heinlein

SUPPORTING INFORMATION:

***Supporting Figures:***

**
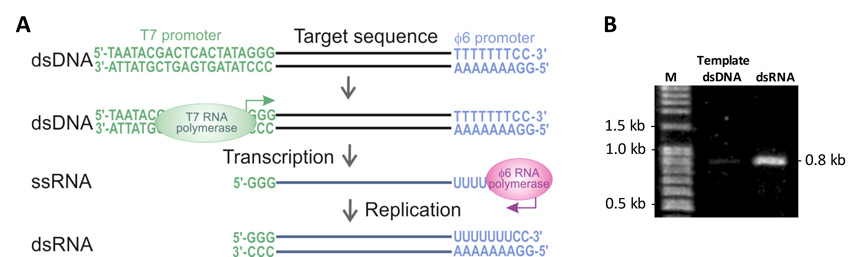
**

**Fig S1:** *In vitro* production of dsRNA. (**A**) dsRNA is enzymatically synthesized from template DNA using T7 DNA-dependent RNA polymerase for transcription and phi6 RNA-dependent RNA polymerase for second-strand RNA synthesis (replication). (**B**) Example of *in vitro* produced λ-phage specific dsRNA^λ^ (0.8 kb). The amount of dsRNA^λ^ shown in the right lane of the agarose gel represents one tenth of the dsRNA produced from 50 ng template DNA (middle lane).


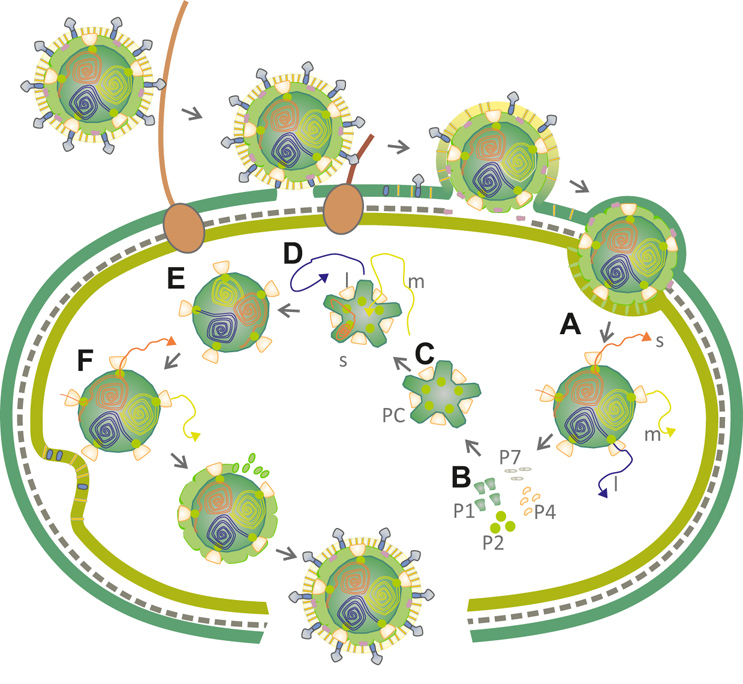


**Fig. S2:** The phi6 lifecycle. (**A**) At the beginning of the phi6 infection the polymerase complex (PC) that forms the internal layer of the phi6 phage particle is delivered in the cytoplasm of the host bacterium. The polymerase subunit residing inside the PC starts the synthesis of phage mRNAs using the encapsidated genomic dsRNA molecules, S, M and L, as templates. These mRNAs (s, m, and l) are delivered into the cytoplasm (**B**) where the L-segment specific ssRNAs (l) directs the synthesis of the proteins P1, P2, P4 and P7. (**C**) These proteins self-assemble into empty PCs, each composed of 120 subunits of the major capsid protein P1, approximately 12 copies of the RNA-dependent RNA polymerase P2, 72 copies of the packaging NTPase protein P4 and approximately 45 copies of the assembly cofactor P7. (**D**) The empty PCs package the phage-specific ssRNA molecules in the order of s, m and l. The specific recognition of each ssRNA is mediated by the unique packaging signals residing at the 5’-terminus of each segment. The energy for the packaging is provided by P4, which is a hexameric helicase residing at the icosahedral five-fold symmetry axes of the PC. (**E**) After completion of the ssRNA packaging the polymerase subunit within the PC is activated and synthetizes the complementary strands for each encapsidated ssRNA segment. The minus-strand synthesis depends on specific RNA secondary structures present in the 3’-termini of the three RNA molecules. (**F**) Subsequently, the polymerase uses the produced dsRNA molecules as templates for semiconservative transcription of the three genomic segments. The produced ssRNA are delivered into the host cytoplasm where they direct the synthesis of the late phage proteins and the subsequent assembly and maturation of the phi6 phage particles.


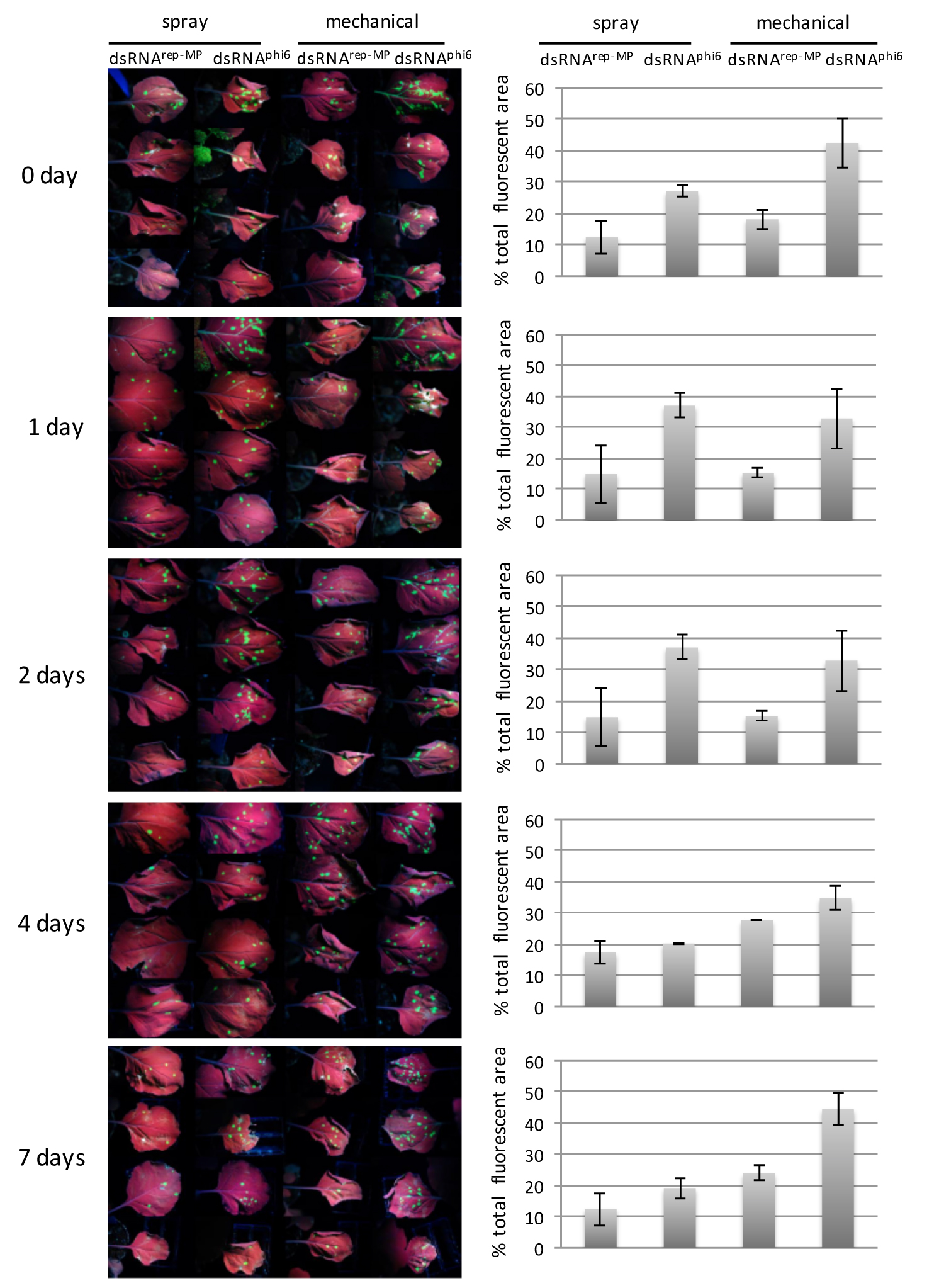


**Fig. S3:** Duration of the protective effect after spraying or mechanical inoculation of dsRNA. Plants were inoculated with *in vivo* produced dsRNA^rep-MP^ or dsRNA^phi6^ by spraying or mechanical inoculation as indicated. TMV-GFP was inoculated to the same leaves at the time of dsRNA inoculation (day 0) or 1, 2, 4, or 7 days later. Four plants per time point of specific dsRNA treatment are shown. Photographs of the virus-inoculated leaves were taken at 7 dpi under UV light (left panel). Quantitative analysis of the relative sizes of the infected leaf areas per condition is shown on the right. The total GFP-fluorescent measured for all the leaves inoculated with GFP-TMV at a certain time point and imaged at 7 dpi was set to 100%, and the distribution of the fluorescence between the conditions was quantified from the images (shown on left). Similar quantitative analysis was also done using images taken at 9 dpi and 14 dpi. The columns show the mean value and standard deviation for these three individual measurements. The figure shows that dsRNA^rep-MP^ provides protection upon spraying or mechanical inoculation for (at least) 7 dpi. A degree of antiviral protection also seen with dsRNA^Phi6^ at later time points may be due to dsRNA-triggered pattern-triggered immunity (PTI) (Niehl et al., 2016). The day 0 plants were also part of the experiment shown in Fig. 5.

S***upporting Tables:***

**Table S1.** Effect of different dsRNAs compared to water on TMV-GFP infection in infected leaves.

|  | **dpi^a^** | **N^b^** | **n^c^** | **% infection^d^** | **p-value^e^** |
| --- | --- | --- | --- | --- | --- |
| TMV-GFP + H_2_O | 7 | 2 | 8 | 100 ± 61.9 | - |
| TMV-GFP | 7 | 1 | 4 | 130.1 ± 63.2 | 0.38 |
| TMV-GFP + dsRNA^λ^ | 7 | 1 | 3 | 56.4 ± 43.8 | 0.12 |
| **TMV-GFP + dsRNA^GFP4^** | 7 | 2 | 8 | 20.4 ± 19.9 | **7.7×10^-6^** |
| **TMV-GFP + dsRNA^GFPv^** | 7 | 2 | 8 | 10.5 ± 9.6 | **2.8×10^-7^** |
| **TMV-GFP + dsRNA^rep5’^** | 7 | 2 | 8 | 28.3 ± 27.2 | **6.9×10^-4^** |
| **TMV-GFP + dsRNA^rep3’^** | 7 | 2 | 8 | 10.0 ± 9.5 | **1.1×10^-5^** |

^a^ dpi, days post infection.

^b^ N, number of independent experiments.

^c^ n, total number of plants per treatment.

^d^ Average infected (GFP-fluorescent) leaf areas in % and their standard deviations. The average infected area of water-treated leaves was set to 100%.

^e^ The p-value (Student’s t-test) determines the statistical difference in infected leaf areas between dsRNA-treated plants and water-treated control plants. Bold letters indicate experiments with significant p-values.

**Table S2.** Effect of different dsRNAs compared to water on the systemic spread of TMV-GFP.

|  | **dpi** ^a^ | **N** ^b^ | **n** ^c^ | **% infection** ^d^ | **p-value** ^e^ |
| --- | --- | --- | --- | --- | --- |
| TMV-GFP + H_2_O | 7 | 2 | 8 | 100 ± 37.9 | - |
| TMV-GFP | 7 | 1 | 4 | 97.3 ± 23.9 | 0.90 |
| TMV-GFP + dsRNA^λ^ | 7 | 1 | 3 | 90.2 ± 33.3 | 0.80 |
| **TMV-GFP + dsRNA^GFP4^** | 7 | 2 | 8 | 10.7 ± 16.0 | **3.5×10^-5^** |
| **TMV-GFP + dsRNA^GFPv^** | 7 | 2 | 8 | 4.4 ± 7.9 | **9.0×10^-6^** |
| **TMV-GFP + dsRNA^rep5^’** | 7 | 2 | 8 | 24.6 ± 27.9 | **4.7×10^-4^** |
| **TMV-GFP + dsRNA^rep3’^** | 7 | 2 | 8 | 8.6 ± 15.5 | **1.9×10^-5^** |

^a^ dpi, days post infection.

^b^ N, number of independent experiments.

^c^ n, total number of plants per treatment.

^d^ Average infected (GFP fluorescent) leaf areas in % and their standard deviations. The average infected area of water-treated leaves was set to 100%.

^e^ The p-value (Student’s t-test) determines the statistical difference in infected leaf areas between dsRNA-treated plants and water-treated control plants. Bold letters indicate experiments with significant p-values.

**Table S3.** Plasmids used for the transformation of *Pseudomonas syringae*.

|  | Plasmid | Description | Name and expected size of the dsRNA |
| --- | --- | --- | --- |
| S-segment  specific | pMH4 | phi6 S-segment in which the lysozyme gene (*gene 5*) has been inactivated | S_lys_, 2948 bp |
|  | pLD18-5 rep-MP | Nucleotides 2850 to 5460 of the TMV genome (replicase and MP gene-specific sequences) cloned into the multiple cloning site of pLD18 | S_TMV_, 3268 bp |
| M-segment specific | pLM656 | phi6 M-segment in plasmid pT7T319U | M, 4063 bp |
|  | pMS2-9 rep-MP | Nucleotides 1921 to 5460 of the TMV genome (replicase and MP gene-specific sequences) cloned into the multiple cloning site of pMS2 | M_TMV_, 4223 bp |
| L-segment specific | pLM991 | phi6 L-segment with kanamycin resistance gene | L_kan_, 7599 bp |

**Table S4.** PCR-primers and other oligonucleotides.

| Oligonucleotide | Sequence | Explanation |
| --- | --- | --- |
| pPS9-MCS_fwd | 5’_CCCGCGGCCGCTAGCATATGTACACCGGTACCCGGGCCCTAGGCCTCGAGCTCGCGATATCGATTAATTAAGCTTGTTTAAACGAATTCTGCAGCCCC_3’ ^1^ | MCS for pLD18; complementary to pPS9-MCS_rev |
| pPS9-MCS_rev | 5’_GGGGCTGCAGAATTCGTTTAAACAAGCTTAATTAATCGATATCGCGAGCTCGAGGCCTAGGGCCCGGGTACCGGTGTACATATGCTAGCGGCCGCGGG_3’ ^1^ | MCS for pLD18; complementary to pPS9-MCS_fwd |
| M_MCS_Sense_AflII_PstI | 5’_**TTAA**CGGCCGCTAGCATATGTACACCGGTACCCGGGCCCTAGGCCTCGAGCTCGCGATATCGATTAATTAAGCTTGTTTAAACGAATTCTGCAG**TGCA**_3’ ^2^ | MCS for pMS1; complementary to M_MCS_antisense |
| M_MCS_antisense | 5’_CTGCAGAATTCGTTTAAACAAGCTTAATTAATCGATATCGCGAGCTCGAGGCCTAGGGCCCGGGTACCGGTGTACATATGCTAGCGGCCG_3’ | MCS for pMS1; complementary to M_MCS_Sense_AflII_PstI |
| PacI_pPS20_3083-3113_FWD | 5’_AAATTTAATTAAGCTTGTTTAAACGAATTCTGCAG_3’ | PCR primers for insertion of the T7 termination sequence into pMS1 to produce pMS2 |
| XbaI_NotI_T7term_pPS20_3343-3323_REV | 5’_CCCCTCTAGAGCGGCCGCCGACAAACAACAGATAAGAGAGAGAGCCCCCGAAGG_3’ |  |
| GFPdsFw | TAATACGACTCACTATAGGG atgagtaaag gagaagaact^3^ | Primers to create the DNA template used to produce GFPv- and GFP4-specific dsRNAs *in vitro* |
| GFPdsRv | GGAAAAAAAttatttgtatagttcatccatg^4^ |  |
| TMV126repfw | TAATACGACTCACTATAGGGatggcataca cacagacagc^3^ | Primers to create the template for *in vitro* production of dsRNA^rep5’^ encompassing nts 1-2000 of the 163k/183k replicase gene |
| TMV126reprv2000 | GGAAAAAAAtaggacgactccggatgatc^4^ |  |
| TMV183fw2851 | TAATACGACTCACTATAGGGcattttgcca aattggaagt^3^ | Primers to create the template for *in vitro* production of dsRNA^rep3’^ encompassing nts 2851-4851 of the replicase gene |
| TMV183revend | GGAAAAAAAttaacaactagagccatctat^4^ |  |

^1^ The *Pst*I and *Eag*I sites are underlined with solid and dashed lines, respectively.

^2^ The 5’ overhang complementary to the cohesive end of the *Afl*II restriction product and the 3’ overhang complementary to the cohesive end of the *Pst*I restriction product are in bold.

^3^ The T7 promoter sequence is shown with uppercase letters and the target sequence with lower case letters.

^4^ The phi6 promoter sequence is shown with uppercase letters and the target sequence with lower case letters.
